# Impact of combining the progesterone receptor and preoperative endocrine prognostic index (PEPI) as a prognostic factor after neoadjuvant endocrine therapy using aromatase inhibitors in postmenopausal ER positive and HER2 negative breast cancer

**DOI:** 10.1371/journal.pone.0201846

**Published:** 2018-08-06

**Authors:** Sasagu Kurozumi, Hiroshi Matsumoto, Kenichi Inoue, Katsunori Tozuka, Yuji Hayashi, Masafumi Kurosumi, Tetsunari Oyama, Takaaki Fujii, Jun Horiguchi, Hiroyuki Kuwano

**Affiliations:** 1 Division of Breast Surgery, Saitama Cancer Center, Saitama, Japan; 2 Department of General Surgical Science, Gunma University Graduate School of Medicine, Gunma, Japan; 3 Division of Breast Oncology, Saitama Cancer Center, Saitama, Japan; 4 Department of Pathology, Saitama Cancer Center, Saitama, Japan; 5 Department of Diagnostic Pathology, Gunma University Graduate School of Medicine, Gunma, Japan; 6 Department of Breast Surgery, International University of Health and Welfare, Chiba, Japan; Florida International University, UNITED STATES

## Abstract

The preoperative endocrine prognostic index (PEPI) predicts survival after neoadjuvant endocrine therapy (NAE) using aromatase inhibitors (AIs) for women with postmenopausal estrogen receptor (ER)-positive breast cancer irrespective of the human epidermal growth factor receptor 2 (HER2) status. Although the progesterone receptor (PgR) is also a prognostic factor for ER-positive breast cancer, the PgR status was not considered a prognostic factor in the original PEPI scoring system. In this study, we investigated the utility of a modified PEPI including the PgR status (PEPI-P) as a prognostic factor after NAE for postmenopausal patients with ER-positive and HER2-negative breast cancer. We enrolled 107 patients with invasive ER-positive and HER2-negative breast cancer treated with exemestane for ≥4 months as NAE. We initially assessed PEPI and compared survival between the groups. Additionally, we obtained an effective cutoff for PgR through survival analysis. Then, we assessed the survival significance of PEPI-P. A PgR staining rate of 50% was the most significant cutoff for predicting recurrence-free survival (RFS) and cancer-specific survival (CSS). PEPI was a significant prognostic factor; moreover, PEPI-P was the most significant prognostic indicator for RFS and CSS. PEPI-P is a potent prognostic indicator of survival after NAE using AIs for postmenopausal patients with ER-positive and HER2-negative breast cancer. This modified PEPI may be useful for therapeutic decision-making regarding postmenopausal ER-positive and HER2-negative breast cancer after NAE.

## Introduction

In both clinical studies and routine practice, neoadjuvant endocrine therapy (NAE) is considered a valid therapeutic option for postmenopausal patients with operable estrogen receptor (ER)-positive breast cancer. Reportedly, NAE enhances the surgical outcomes of both early cases of operable breast cancer in which breast-conserving surgery is desired and advanced cases in which mastectomy is technically impossible [1–5]. Using aromatase inhibitors (AIs), NAE has proven to effectively decrease tumor size in postmenopausal patients with ER-positive breast cancer. In the St. Gallen consensus meeting, NAE without chemotherapy was suggested as a practical option for postmenopausal patients with endocrine therapy-responsive tumors [6].

To assess patients’ outcomes, Ellis et al. [7] proposed a scoring system, the preoperative endocrine prognostic index (PEPI), consisting of the pathological tumor size, pathological node status, Ki67 labeling index, and ER status of residual tumors after NAE. The efficacy of the PEPI scoring system for estimating recurrence-free survival (RFS) in patients receiving NAE has been established. Recently, a study illustrated that the progesterone receptor (PgR) status can indicate a poor prognosis in patients with ER-positive and human epidermal growth factor receptor 2 (HER2)-negative breast cancer [8]. However, because the PgR status was not included in the original PEPI scoring system, the utility of PgR expression in primary tumors (p-PgR) as a prognostic factor for patients who received NAE remains unclear.

Hence, this study compared the original PEPI to a modified p-PgR status alone and a modified PEPI including the PgR status (PEPI-P) as a prognostic factor for NAE in postmenopausal patients with ER-positive and HER2-negative breast cancer.

## Patients and methods

### Background and eligibility of patients

In this study, we enrolled 107 postmenopausal Japanese women with ER-positive and HER2-negative invasive breast cancer who received NAE using exemestane and underwent surgery at Saitama Cancer Center Hospital (Saitama, Japan) between 2003 and 2013. According to the ER, PgR, and HER2 statuses, exemestane (25 mg/day) was preoperatively administered to all patients with ER-positive and HER2-negative breast cancer for 4–58 (median, 6) months. Additionally, NAE using exemestane was administered to increase the breast conservation rate by decreasing tumor size, and the histopathological response to exemestane therapy was assessed. Then, consecutive AIs or tamoxifen was administered as an adjuvant therapy to 106 (99.1%) patients, and only 1 (0.9%) patient received no adjuvant endocrine therapy. This study protocol was approved by the Institutional Review Board of the Saitama Cancer Center (reference number 484), and it adhered to the tenets of the Declaration of Helsinki. Furthermore, we obtained comprehensive written informed consent for the scientific examination of tumor specimens resected via biopsy and surgery.

### Evaluation of histopathological responses

We estimated histopathological responses to preoperative endocrine therapy using the grading system established by the Japanese Breast Cancer Society (JBCS). In this system, pathological effects are defined using grades of 0, 1a, 1b, 2a, 2b, and 3 according to morphological damage assessed via the extent of tumor cell degeneration or disappearance of the invasive tumor. Specifically, grades 0–1a comprised the “mild” pathological response group, and grades 1b–3 comprised the “marked” pathological response group. Grade 1a was characterized by “mild changes in cancer cells without the regard of extent, and/or marked changes of the tumor in <1/3 of area,” and grade 1b was characterized by “marked changes recognized in 1/3–2/3 of the tumor.” Grade 2a was characterized by “marked changes found in ≥2/3 of the tumor, but remaining the viable cells,” whereas grade 2b was characterized by “marked changes near complete response with only a few remaining cancer cells.” Finally, grade 3 was considered a “pathological complete response (pCR)” as proposed in the NSABP B-18 study [9] and characterized as “no invasive cancer in the breast”.

### Procedures and evaluation of immunohistochemistry and dual *in situ* hybridization (DISH)

In this study, we used buffered formalin-fixed, paraffin-embedded tumor tissues obtained via needle biopsy before NAE and via surgery after NAE. The immunohistochemical assays for ER, PgR, HER2, and Ki67 and DISH examination have been described in detail elsewhere [8]. Additionally, our previous study presented methods of assessing the ER, PgR, HER2, and Ki67 statuses [8].

Briefly, we determined the proportions of ER- and PgR-positive cells by counting the number of positive nuclei, with ≥1% positive cells adjudged as “positive.” Additionally, the ER and PgR staining rates were determined using the Allred score. We defined “20%” and “50%” as cutoff points for PgR positivity according to previous research [8]. The HER2 status was established according to the results of immunohistochemistry and DISH using the 2013 ASCO/CAP guidelines. Finally, we calculated the number of Ki67-positive and Ki67-negative nuclei among approximately 500 tumor cells in warm to hot areas and determined the labeling index.

### Statistical analysis

We performed statistical analysis using SPSS v24.0 (IBM Corp., Armonk, NY, USA). Additionally, we used the Kaplan–Meier method and log-rank test to estimate RFS and cancer-specific survival (CSS). Of note, RFS was defined as the time from the start of NAE to any recurrence, and CSS was defined as the time from the start of NAE to death due to breast cancer. We compared the survival rates of two groups divided by the extent of p-PgR staining and determined the most appropriate cutoff values for identifying patients with poor prognosis. Also, RFS and CSS in the two patient groups stratified by pathological tumor size (ypT1/2 vs. ypT3/4), pathological node status (ypN negativity vs. ypN positivity), pathological stage after NAE (r-stage 1/2 vs. r-stage 3/4), pathological response (marked pathological responses vs. mild pathological responses), and surgical specimen ER (r-ER) status (Allred score ≥ 3 vs. 0 or 2) were compared.

In this study, all patients were divided into five groups based on the Ki67 labeling index of residual tumors after NAE (r-Ki67) as follows: (a) r-Ki67, 0%–2.7%; (b) r-Ki67, 2.7%–7.3%; (c) r-Ki67, 7.3%–19.7%; (d) r-Ki67, 19.7%–53.1%; and (e) r-Ki67, >53.1% [7]. RFS and CSS were analyzed using this r-Ki67 classification. Furthermore, we evaluated PEPI (scored 0–12) in each patient based on the statuses of four factors (ypT, ypN, r-ER, and r-Ki67) according to the PEPI-P scoring system (S1 Table). Of note, we excluded 7 (6.5%) patients from the PEPI evaluation because they received no axillary lymph node management (5 patients), they achieved pCR (1 patient), or it was impossible to evaluate paraffin-embedded surgical specimens for the r-Ki67 labeling index (1 patient). Furthermore, PEPI was classified into three risk groups as follows: low (0 points), moderate (1–3 points), and high (≥4 points). RFS and CSS were compared among these three PEPI groups.

Moreover, we included the p-PgR status in PEPI-P and assessed the new scoring system. Tumors with ≥50% p-PgR staining were assigned to the high p-PgR group according to the highest hazard ratio (HR) derived from the survival analysis, whereas tumors with <50% p-PgR staining were placed in the low p-PgR group. We defined the PgR score as “low PgR expression was score 3 and high PgR expression was score 0” according to the HR obtained via univariate analysis. Furthermore, we analyzed RFS and CSS in the three PEPI-P risk groups as follows: low (0–3), moderate (4–6), and high (≥7). Differences with *P* < 0.05 were considered significant.

## Results

### Characteristics of patients and tumors

S2 Table summarizes the characteristics of patients and tumors in this study. The median age of the 107 patients enrolled in this study was 65 (range, 49–84) years. In total, 5.6% of patients had clinical T3 or T4 tumors, and 15.0% of patients had a clinical lymph node metastasis-positive status. Overall, 19 (17.8%) patients received adjuvant chemotherapy. S3 Table presents the distribution of patients stratified by the ypT and ypN statuses. The results illustrated that 6.6% of patients were categorized as ypT3 or ypT4, and 41.1% of patients had a ypN-positive status. Regarding the tumor response to NAE, 50 of 107 (46.7%) patients exhibited significant responses (grades 1b–3, JBCS criteria). The histological tumor responses of all patients were as follows: grade 3 (pCR), 1 patient (0.9%); grade 2b, 1 patient (0.9%); grade 2a, 8 patients (7.5%); grade 1b, 40 patients (37.4%); and grade 0–1a, 57 patients (53.3%; S3 Table).

### Distribution of the PgR status and survival according to the p-PgR status

**Fig 1:**
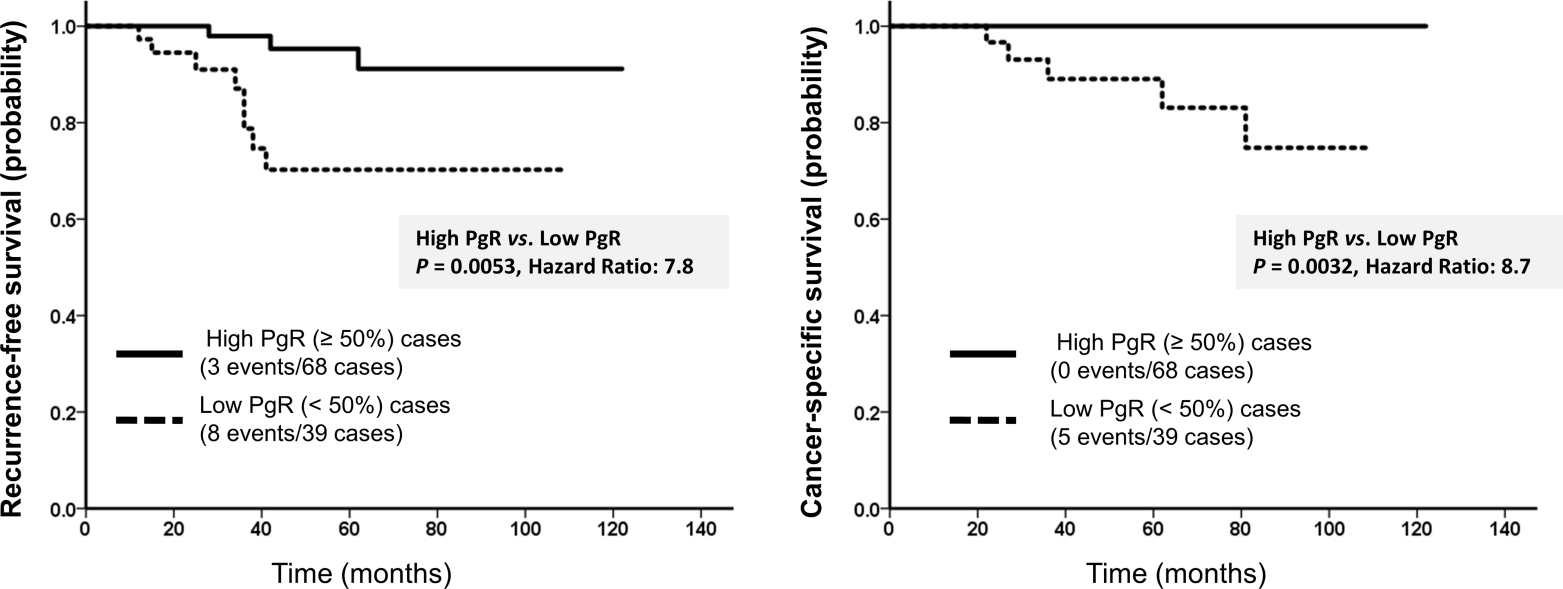
Recurrence-free survival (RFS) and cancer-specific survival (CSS) curves for the high (≥50%) and low primary progesterone receptor (p-PgR) (<50%) groups. RFS and CSS were significantly better in the high p-PgR group than in the low p-PgR group (RFS: *P* = 0.0053; CSS: *P* = 0.0031).

S4 Table describes the distribution of p-PgR expression stratified by Allred scores and p-PgR expression. The p-PgR positivity rate was 82.2% (88 patients). We observed no correlation between p-PgR expression and PEPI (*P* = 0.075). Table 1 presents the HRs of RFS and CSS stratified by the p-PgR status. Concerning differences between the low and high p-PgR expression groups stratified by Allred scores regarding the probability of survival, the most significant cutoff point was 7 (RFS: HR = 8.7, *P* = 0.0032; CSS: HR = 4.8, *P* = 0.028). Also, regarding the differences between the high and low p-PgR expression groups stratified by the percentage of positive cells concerning the probability of survival, a p-PgR positivity rate of 50% was the most suitable cutoff point for prognosis (RFS: HR = 7.8, *P* = 0.0053; CSS: HR = 8.7, *P* = 0.0031; Fig 1).

**Table 1:** Survival analysis according to the Allred score and PgR staining percentage in primary tumors.

Allred scores for PgR	RFS		CSS	
Cutoff point	HR	*P*-value	HR	*P*-value
0 vs. 2–8	2.2	0.14	2.2	0.13
0–2 vs. 2–8	6.0	0.014	1.2	0.27
0–3 vs. 2–8	1.3	0.25	0.2	0.69
0–4 vs. 2–8	4.6	0.0033	1.0	0.31
0–5 vs. 2–8	4.9	0.027	1.3	0.25
0–6 vs. 2–8	8.7	0.0032	4.8	0.028
0–7 vs. 2–8	4.1	0.042	1.6	0.21
PgR positivity rate	RFS		CSS	
Cutoff point	HR	*P*-value	HR	*P*-value
0	2.2	0.13	2.2	0.13
1	0.9	0.34	0.09	0.76
10	4.8	0.029	5.2	0.022
20	6.3	0.012	3.4	0.065
33	5.5	0.019	4.8	0.028
50	7.8	0.0053	8.7	0.0031
67	4.0	0.046	2.6	0.11

Abbreviations: PgR, progesterone receptor; RFS, Recurrence-free survival; CSS, Cancer-specific survival; HR, Hazard ratio.

### Distribution of patients and survival analysis stratified by the PgR status and Ki67 labeling index in residual tumors

S4 Table presents the distribution of patients stratified by the r-Ki67 labeling index. Table 2 shows the HRs for RFS and CSS according to the r-Ki67 labeling index. In this study, the r-Ki67 classification was correlated with RFS but not CSS (RFS: HR = 10.6, *P* = 0.032; CSS: HR = 5.2, *P* = 0.27). S5 Table presents the distribution of patients stratified by r-PgR. Furthermore, 64 (60.4%) patients had r-PgR-negative breast cancer, and the PgR status in residual tumors was not a significant prognostic factor for NAE (S6 Table).

**Table 2:** Survival analysis of the influence of clinicopathological variables including the preoperative endocrine prognostic index (PEPI) and PEPI combined with the primary PgR status (PEPI-P).

Factors	*Number*	RFS		CSS	
		HR	*P*-value	HR	*P*-value
Residual tumor size					
ypT1/2	100	Reference			
ypT3/4	7	14.4	0.00015	4.4	0.036
Residual node status					
Negative	58	Reference			
Positive	44	12.9	0.00033	6.3	0.012
Residual Ki67 level					
0–2.7%	35	Reference			
>2.7–7.3%	18	0.3	0.6	1.7	0.19
>7.3–19.7%	29	3.7	0.054	2.4	0.13
>19.7–53.1%	20	3.4	0.066	1.3	0.25
>53.1%	4	10.7	0.0011	6.8	0.0094
Total	106	10.6	0.032	5.2	0.27
Residual ER Allred score					
3–8	106	Reference			
0–2	0	NE	NE	NE	NE
PEPI					
0	25	Reference			
1–3	35	NE	NE	NE	NE
≥4	40	6.2	0.013	2.9	0.092
Primary PgR score					
≥50%	68	Reference			
<50%	39	7.8	0.0053	8.7	0.0032
PEPI-P					
0–3	49	Reference			
4–6	31	2.6	0.11	NE	NE
≥7	20	20.3	<0.00001	10.8	0.001

Abbreviations: PgR, progesterone receptor; RFS, Recurrence-free survival; CSS, Cancer-specific survival; HR, Hazard ratio; ER, estrogen receptor; NE, not evaluated.

### Survival analysis according to clinicopathological characteristics including PEPI and PEPI-P scores

**Fig 2:**
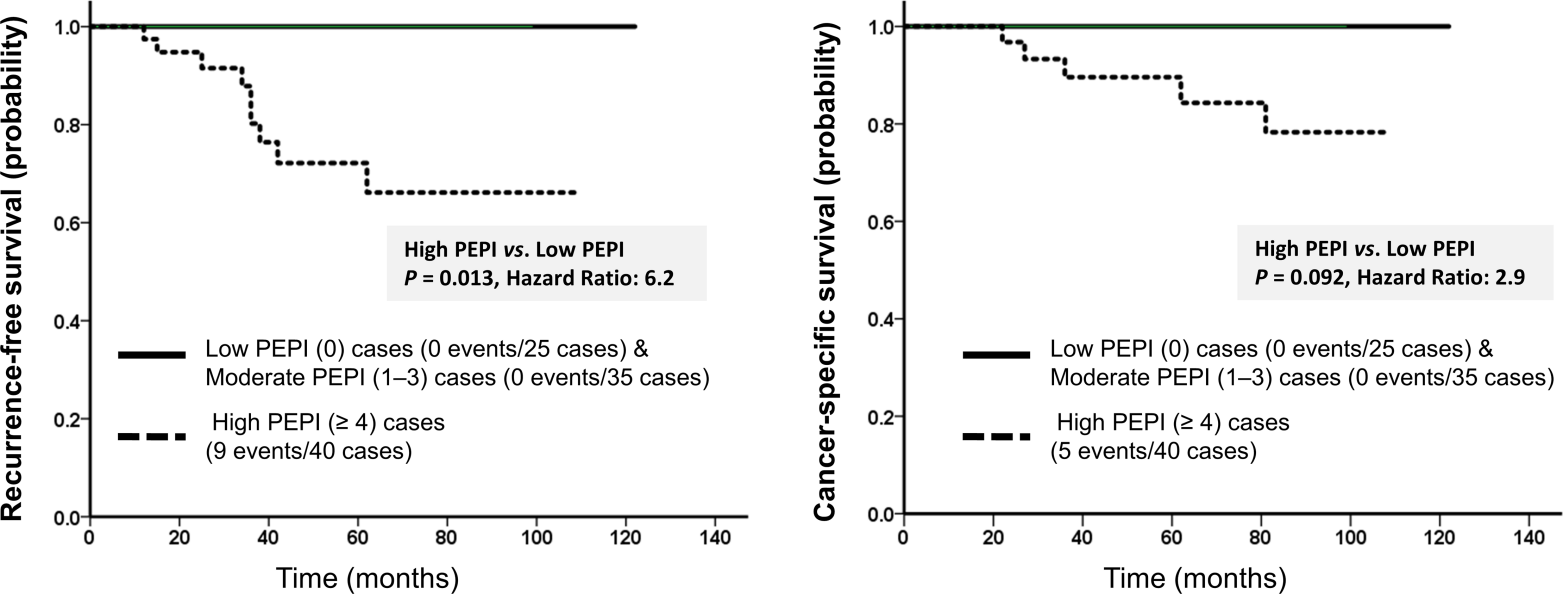
Cumulative survival of patients with breast cancer stratified by the original preoperative endocrine prognostic index (PEPI). (A) Recurrence-free survival (RFS) was significantly worse in the high PEPI group than in the low PEPI group (*P* = 0.013). (B) No significant differences were noted in cancer-specific survival (CSS) between the high and low PEPI groups (*P* = 0.092).

In this study, survival was better in the marked response group than in the mild response group, but the differences were not statistically significant (RFS: HR = 3.5, *P* = 0.060; CSS: HR = 1.1, *P* = 0.29). According to univariate analysis (Table 2), low p-PgR expression (RFS: HR = 7.8, *P* = 0.0053; CSS: HR = 8.7, *P* = 0.0031), a high r-Ki67 labeling index (RFS: HR, 10.6, *P* = 0.032; CSS: HR, 5.2, *P* = 0.27), high ypT (RFS: HR, 14.4, *P* = 0.00015; CSS: HR, 4.4, *P* = 0.036), and ypN positivity (RFS: HR = 12.9, *P* = 0.00033; CSS: HR = 6.3, *P* = 0.012) indicated worse prognosis. Table 2 presents the HRs for RFS and CSS stratified by PEPI. RFS was significantly different between high and low PEPI groups (Fig 2).

**Fig 3:**
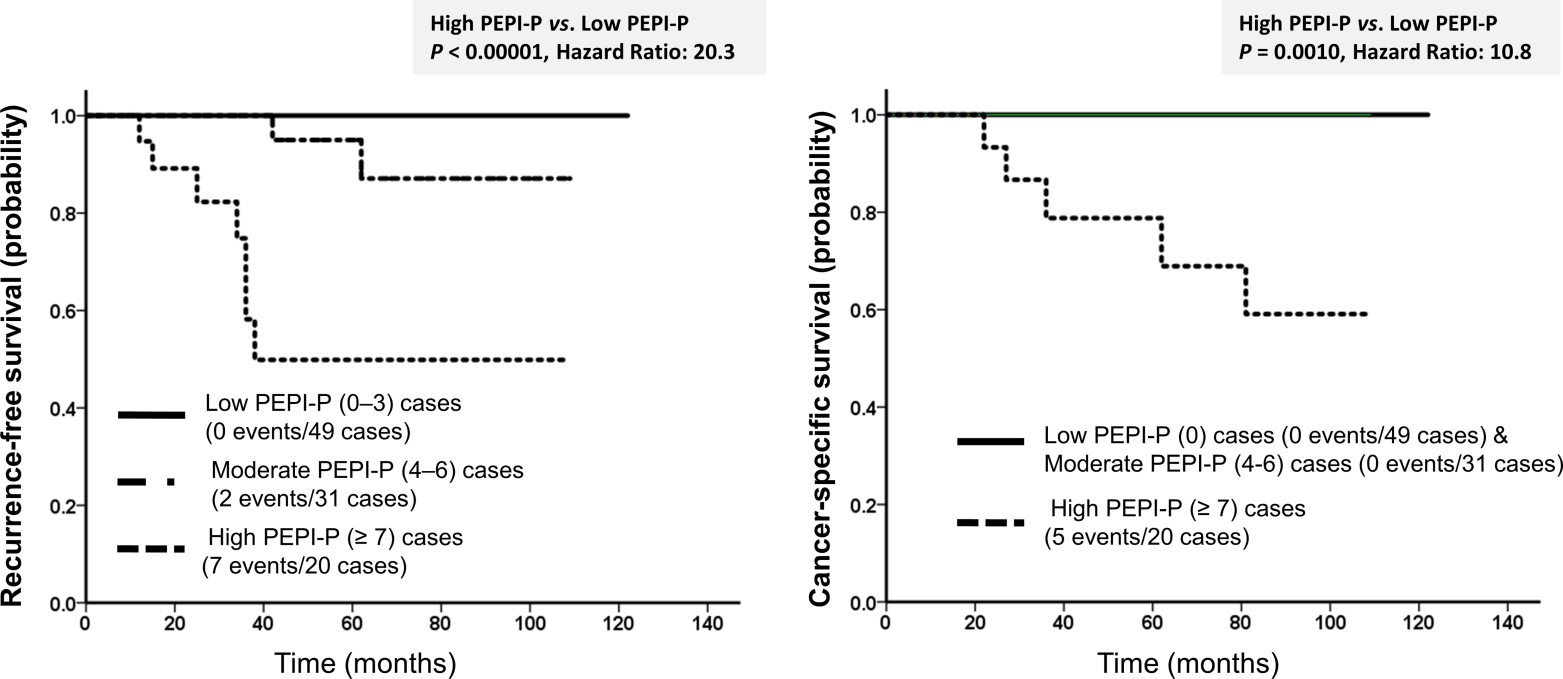
Cumulative survival of patients with breast cancer stratified by our modified PEPI including the primary PgR status (PEPI-P). Relationships of PEPI-P with (A) recurrence-free (RFS) and (B) cancer-specific survival (CSS).

Additionally, the distribution of patients according to PEPI-P for RFS and CSS was as follows: (a) low (scores 0–3), 49 (49.0%) patients; (b) intermediate (scores 4–6), 31 (31.0%) patients; and (c) high (score ≥7), 20 (20.0%) patients. Table 2 shows the HRs for RFS and CSS stratified by PEPI-P. PEPI-P was the most significant prognostic indicator for both RFS and CSS (Fig 3).

## Discussion

This study revealed that p-PgR expression was a significant prognostic factor for NAE using exemestane, and the best cutoff for p-PgR was 50%. Also, PEPI was a potent prognostic tool, especially for RFS. Thus, our new system combining the p-PgR index with PEPI was a significant prognostic indicator for survival in this NAE setting.

PgR is a crucial factor in the progression of ER-positive breast cancer because it controls the expression of various genes and proteins through the genomic pathway [10]. In this pathway, PgR mediates intracellular estrogen/progesterone-related signaling pathways as a regulator of transcription for target genes in conjunction with other transcription factors such as signal transducer and activator of transcription [11], specificity protein 1 [12], and activator protein 1 [13]. Conversely, PgR-related signaling pathways are critically involved in the maintenance of breast cancer phenotypes. Mohammed et al. inferred that genomic variations in *PgR* represent a typical mechanism for decreases of PgR protein expression, which might induce alterations in the expression patterns of the ER-binding gene, affecting breast tumor proliferation and explaining the poor outcomes of patients with luminal breast cancer [14]. Some studies reported that the non-genomic pathway was activated through signal crosstalk between estrogen/progesterone and several growth factors [15, 16]. As the mechanism for the development of ER-positive, PgR-negative breast cancers, the downregulation of PgR expression might be correlated with crosstalk with epidermal growth factor receptor pathways, such as human epidermal growth factor receptor 1 and insulin-like growth factor 1 signaling [17, 18].

PgR expression was considered to be related to the response to tamoxifen, and several retrospective studies including our previous study indicated that PgR expression according to the Allred score was a significant prognostic factor in ER-positive, HER2-negative breast cancer in patients treated with tamoxifen [8, 19]. In several clinical trials, PgR was not predictive of the pathological response to NAE with AIs [20, 21]. NAE using AIs is a rational treatment option for postmenopausal patients with ER-positive breast cancer [22]. Dowsett et al. reported that the tumor diameter, ER expression, and the Ki67 labeling index at baseline, as well as the r-ER status and r-Ki67 labeling index after NAE, were significantly predictive of RFS [20]. Additionally, Ellis et al. reported that the ypT, ypN, r-Ki67 labeling index, and r-ER statuses after the initiation of NAE using letrozole or tamoxifen significantly predicted RFS and CSS. Although clinical tumor responses and histological grades also predicted RFS, these factors were not significant in a multivariate analysis [7, 23]. Thus, the ypT, ypN, r-ER, and r-Ki67 labeling index statuses are standard prognostic and predictive markers of breast cancer in patients treated with NAE. Furthermore, PEPI, which comprises these factors, was also identified as a potent prognostic factor for patients with ER-positive breast cancer [7].

According to the 2017 St. Gallen consensus meeting, adjuvant treatments should be escalated or de-escalated on the basis of patient benefit, considering the tumor biology and patient prognosis, in addition to the intensity, duration, and side effects of the adjuvant treatments [24]. Typically, ER–positive and HER2-negative breast cancer can be divided into luminal A-like, luminal B-like, and intermediate subtypes according to the pathological characteristics [6]. This consensus guideline recommends that adjuvant chemotherapy is de-escalated for patients with luminal A-like breast cancer and escalated for those with luminal B-like breast cancer [24]. However, adjuvant treatment strategies for patients with intermediate breast cancer, who comprise >50% of all patients with ER-positive, HER2-negative breast cancer, remain undefined [8]. To increase the frequency of de-escalation of chemotherapy among patients with ER-positive and HER2-negative breast cancer, the population of the intermediate group should be reduced. In the initial trial of PEPI [7], approximately 15%–25% of cases had a PEPI of 0, suggesting that chemotherapy could be omitted. Another study reported that PEPI could be a potent tool for selecting NAE-treated patients with ER–positive and HER2-negative breast cancer of the luminal A-like subtype [25]. In present result, 25% cases were PEPI score 0 and 49% cases had low PEPI-P score. Furthermore; the cancer specific survival was significant different between for low & intermediate groups and for high group stratified by PEPI-P score, and 80% cases were low & intermediate PEPI-P scoring groups.Our findings suggest that PEPI-P could be a potent tool for therapeutic decision-making regarding NAE-treated patients with ER-positive and HER2-negative breast cancer of the intermediate subtype.

Reportedly, luminal B-like tumors are associated with worse outcomes than luminal A-like tumors even after the administration of hormonal therapy [26]; however, the differences of the mechanisms of survival, proliferation, and metastasis between luminal A- and luminal B-like breast cancers remain unclear [27]. Recent findings indicated that several molecular targeted therapies such as cyclin-dependent kinases 4/6 inhibitors were useful in patients with endocrine therapy-resistant breast cancer [28]. To explore the utility of these new therapies as neoadjuvant treatments, it may be required to identify significant prognostic indicators for predicting overall survival or CSS for NAE-treated patients with ER-positive and HER2-negative breast cancer before conducting prospective clinical trials of new drugs in the neoadjuvant setting. Further translational studies are necessary to investigate the association of PEPI-P with the response to these molecular targeted therapies in patients with ER-positive and HER2-negative breast cancer who are resistance to endocrine treatment.

In conclusion, the survival analysis based on the percentage of PgR staining in residual tumors after NAE suggested that the PgR status in residual tumors is not a significant prognostic factor for NAE. PEPI is a significant prognostic factor for ER-positive and HER2-negative breast cancer. Also, a p-PgR staining rate of 50% is a significant cutoff for predicting the outcomes of NAE-treated patients with ER-positive and HER2-negative breast cancer similarly as PEPI. Thus, PEPI-P, which combines PEPI with the p-PgR status, could be a potent prognostic indicator.

## Supporting information

S1 Table Criteria of the preoperative endocrine prognostic index (PEPI) [7]. (DOCX)

S2 Table Patient and tumor characteristics at baseline. (DOCX)

S3 Table Histological tumor responses after neoadjuvant endocrine therapy. (DOCX)

S4 Table Distribution of the expression of PgR in primary tumors (p-PgR) and the residual Ki67 (r-Ki67) labeling index. (DOCX)

S5 Table Distribution of residual PgR (r-PgR) expression. (DOCX)

S6 Table Survival analysis according to the Allred score and PgR staining percentage in residual tumors (r-PgR). (DOCX)
