# Supplementary material for: Impact of combining the progesterone receptor and preoperative endocrine prognostic index (PEPI) as a prognostic factor after neoadjuvant endocrine therapy using aromatase inhibitors in postmenopausal ER positive and HER2 negative breast cancer
Source: PLoS One. 2018 Aug 6;13(8):e0201846. doi: 10.1371/journal.pone.0201846 (PMC6078304; doi:10.1371/journal.pone.0201846)
Supplement: S1 Table — (DOCX) [file pone.0201846.s001.docx]

S1 Supporting Information

Criteria of the preoperative endocrine prognostic index (PEPI) [7]

| Preoperative Prognostic Index (PEPI) | | |
| --- | --- | --- |
|  | RFS | CSS |
| Residual Tumor Size |  |  |
| ypT1/2 | 0 | 0 |
| ypT3/4 | 3 | 3 |
| Residual Node Status |  |  |
| Negative | 0 | 0 |
| Positive | 3 | 3 |
| Residual Ki67 Level |  |  |
| 0-2.7% | 0 | 0 |
| >2.7-7.3% | 1 | 1 |
| >7.3-19.7% | 1 | 2 |
| >19.7%-53.1% | 2 | 3 |
| >53.1% | 3 | 3 |
| Residual ER Allred Score |  |  |
| 0-2 | 3 | 3 |
| 3-8 | 0 | 0 |
