# Supplementary material for: Impact of combining the progesterone receptor and preoperative endocrine prognostic index (PEPI) as a prognostic factor after neoadjuvant endocrine therapy using aromatase inhibitors in postmenopausal ER positive and HER2 negative breast cancer
Source: PLoS One. 2018 Aug 6;13(8):e0201846. doi: 10.1371/journal.pone.0201846 (PMC6078304; doi:10.1371/journal.pone.0201846)
Supplement: S2 Table — (DOCX) [file pone.0201846.s002.docx]

S2 Supporting Information

Patient and tumor characteristics at baseline

No. of patients %

Total 107 100

Age range in years

< 60 29 27.1

≥ 60 78 72.9

Clinical tumor size

T1 25 23.4

T2 76 71.0

T3 3 2.8

T4 3 2.8

Clinical nodal status

N0 91 85.0

N1 15 14.0

N2 1 0.9

Clinical stage

I 23 21.5 IIA 66 61.7

IIB 13 12.1

IIIA 2 1.9 IIIB 3 2.8

Type of surgery

Breast-conserving surgery 99 92.5

Mastectomy 8 7.5

Axillary management

Sentinel lymph node biopsy alone 69 64.5

Axillary lymph node dissection 33 30.9

No surgery 5 4.7

Adjuvant Chemotherapy

Yes 19 17.8

No 88 82.2
