# Supplementary material for: Impact of combining the progesterone receptor and preoperative endocrine prognostic index (PEPI) as a prognostic factor after neoadjuvant endocrine therapy using aromatase inhibitors in postmenopausal ER positive and HER2 negative breast cancer
Source: PLoS One. 2018 Aug 6;13(8):e0201846. doi: 10.1371/journal.pone.0201846 (PMC6078304; doi:10.1371/journal.pone.0201846)
Supplement: S3 Table — (DOCX) [file pone.0201846.s003.docx]

S3 Supporting Information

Histological tumor responses after neoadjuvant endocrine therapy

No. of patients %

Total 107 100

Histological Grade

Grade 3 (pCR) 1 0.9

Grade 2b 1 0.9

Grade 2a 8 7.5

Grade 1b 40 37.4

Grade 0-1a 57 53.3

Residual tumor size

ypT0 1 0.9

ypT1 59 55.1

ypT2 40 37.4

ypT3 5 4.7

ypT4 2 1.9

Residual nodal status

ypN0 58 54.2

ypN1 34 31.8

ypN2 7 6.5

ypN3 3 2.8

No surgery 5 4.7
