# Supplementary material for: Impact of combining the progesterone receptor and preoperative endocrine prognostic index (PEPI) as a prognostic factor after neoadjuvant endocrine therapy using aromatase inhibitors in postmenopausal ER positive and HER2 negative breast cancer
Source: PLoS One. 2018 Aug 6;13(8):e0201846. doi: 10.1371/journal.pone.0201846 (PMC6078304; doi:10.1371/journal.pone.0201846)
Supplement: S4 Table — (DOCX) [file pone.0201846.s004.docx]

S4 Supporting Information

Distribution of primary-PgR (p-PgR) expression and the residual Ki67 (r-Ki67) labeling index

Allred Scores of p-PgR No. %

0 12 11.2

2 1 0.9

3 5 4.7

4 5 4.7

5 11 10.3

6 19 17.8

7 17 15.9

8 37 34.6

Proportion of p-PgR (%) No. %

0 12 11.2

0-1 7 6.5

1-10 5 4.7

10-20 5 4.7

20-33 6 5.6

33-50 4 3.7

50-67 31 29.0

>67 37 34.6

r-Ki67 labeling index (%) No. %

0-2.7 35 32.7

2.7-7.3 18 16.8

7.3-19.7 29 27.1

19.7-53.1 20 18.7

≥53.1 4 3.7
