# Supplementary material for: Impact of combining the progesterone receptor and preoperative endocrine prognostic index (PEPI) as a prognostic factor after neoadjuvant endocrine therapy using aromatase inhibitors in postmenopausal ER positive and HER2 negative breast cancer
Source: PLoS One. 2018 Aug 6;13(8):e0201846. doi: 10.1371/journal.pone.0201846 (PMC6078304; doi:10.1371/journal.pone.0201846)
Supplement: S5 Table — (DOCX) [file pone.0201846.s005.docx]

S5 Supporting Information

Distribution of residual PgR (r-PgR) expression

Proportion of r-PgR (%) No. %

0 58 54.7

0-1 6 5.7

1-10 9 8.5

10-50 10 9.4

*>*50 23 21.7
