# Supplementary material for: Impact of combining the progesterone receptor and preoperative endocrine prognostic index (PEPI) as a prognostic factor after neoadjuvant endocrine therapy using aromatase inhibitors in postmenopausal ER positive and HER2 negative breast cancer
Source: PLoS One. 2018 Aug 6;13(8):e0201846. doi: 10.1371/journal.pone.0201846 (PMC6078304; doi:10.1371/journal.pone.0201846)
Supplement: S6 Table — (DOCX) [file pone.0201846.s006.docx]

S6 Supporting Information

Survival analysis according to the Allred score and staining percentage of PgR in residual tumors (r-PgR)

Proportion of r-PgR RFS CSS

Cut-off point (%) HR  *P*  HR *P*

0 0.7 0.42 0.5 0.50

1 0.5 0.50 0.2 0.70

10 0.0 0.99 0.9 0.35

50 0.7 0.42 0.2 0.66

Abbreviations:

RFS, Recurrence-free survival; CSS, Cancer-specific survival; HR, Hazard Ratio; PgR, progesterone receptor.
